# Loss or gain of function? Effects of ion channel mutations on neuronal firing depend on the neuron type

**DOI:** 10.3389/fneur.2023.1194811

**Published:** 2023-05-24

**Authors:** Nils A. Koch, Lukas Sonnenberg, Ulrike B. S. Hedrich, Stephan Lauxmann, Jan Benda

**Affiliations:** ^1^Institute of Neurobiology, Faculty of Mathematics and Natural Sciences, University of Tübingen, Tübingen, Germany; ^2^Bernstein Center for Computational Neuroscience Tübingen, Tübingen, Germany; ^3^Department of Neurology and Epileptology, Hertie Institute for Clinical Brain Research, University of Tübingen, Tübingen, Germany

**Keywords:** channelopathies, epilepsy, ataxia, potassium currents, neuronal simulation, conductance-based models, neuronal heterogeneity

## Abstract

**Introduction:**

Clinically relevant mutations to voltage-gated ion channels, called channelopathies, alter ion channel function, properties of ionic currents, and neuronal firing. The effects of ion channel mutations are routinely assessed and characterized as loss of function (LOF) or gain of function (GOF) at the level of ionic currents. However, emerging personalized medicine approaches based on LOF/GOF characterization have limited therapeutic success. Potential reasons are among others that the translation from this binary characterization to neuronal firing is currently not well-understood—especially when considering different neuronal cell types. In this study, we investigate the impact of neuronal cell type on the firing outcome of ion channel mutations.

**Methods:**

To this end, we simulated a diverse collection of single-compartment, conductance-based neuron models that differed in their composition of ionic currents. We systematically analyzed the effects of changes in ion current properties on firing in different neuronal types. Additionally, we simulated the effects of known mutations in *KCNA1* gene encoding the K_V_1.1 potassium channel subtype associated with episodic ataxia type 1 (EA1).

**Results:**

These simulations revealed that the outcome of a given change in ion channel properties on neuronal excitability depends on neuron type, i.e., the properties and expression levels of the unaffected ionic currents.

**Discussion:**

Consequently, neuron-type specific effects are vital to a full understanding of the effects of channelopathies on neuronal excitability and are an important step toward improving the efficacy and precision of personalized medicine approaches.

## 1. Introduction

The properties and combinations of voltage-gated ion channels are vital in determining neuronal excitability (1–4). However, ion channel function can be disturbed, for instance, through genetic alterations, resulting in altered neuronal firing behavior (2). In recent years, next generation sequencing has led to an increase in the discovery of clinically relevant ion channel mutations and has provided the basis for pathophysiological studies of genetic epilepsies, pain disorders, dyskinesias, intellectual disabilities, myotonias, and periodic paralyzes (1, 2). Ongoing efforts of many research groups have contributed to the current understanding of underlying disease mechanism in channelopathies. However, a complex pathophysiological landscape has emerged for many channelopathies and is likely a reason for limited therapeutic success with standard care.

Ion channel variants are frequently classified in heterologous expression systems as either a loss of function (LOF) or a gain of function (GOF) in the respective ionic current (5–8). This LOF/GOF classification is often directly used to predict the effects on neuronal firing (9–12), which in turn is important for understanding the pathophysiology of these disorders and for identification of potential therapeutic targets (13–16). Experimentally, the effects of channelopathies on neuronal firing are assessed using primary neuronal cultures (17–19) or *in vitro* recordings from slices of transgenic mouse lines (20–24) but are restricted to a limited number of different neuron types. Neuron types differ in many aspects. They may differ in their composition of ionic currents (25–28) and, therefore, likely respond differently to changes in the properties of a single ionic current. The expression level of an affected gene (29) and relative amplitudes of ionic currents (3, 4, 30–32) indeed dramatically influence the firing behavior and dynamics of neurons. Mutations in different sodium channel genes have been experimentally shown to affect firing in a neuron-type specific manner based on differences in expression levels of the affected gene (29) but also on other neuron-type specific mechanisms (24, 33).

Neuron-type specificity is likely vital for successful precision medicine treatment approaches. For example, Dravet syndrome was identified as the consequence of LOF mutations in encoding the voltage-gated sodium channel Nav1.1. *SCN1A* (34–36); however, limited success in the treatment of Dravet syndrome persisted (34, 37) in part due to the lack of understanding that inhibitory interneurons and not pyramidal neurons had altered excitability as a result of LOF *SCN1A* mutations (15, 16).

Taken together, these examples demonstrate the need to study the effects of ion channel mutations in many different neuron types—a daunting if not impossible experimental challenge. In the context of this diversity, simulations of conductance-based neuronal models are a powerful tool bridging the gap between altered ionic currents and firing in a systematic and efficient way. Furthermore, simulations allow to predict the potential effects of drugs needed to alleviate the pathophysiology of the respective mutation (38–40).

In this study, we therefore investigated how the outcome of ionic current kinetic changes on firing depends on neuronal cell type, i.e., on the composition of ionic currents, by (1) characterizing firing responses with two measures, (2) simulating the response of a repertoire of different neuronal models to changes in single current parameters, and (3) bringing more complex changes in this case as they were observed for specific *KCNA1* mutations that are associated with episodic ataxia type 1 (39, 41, 42).

## 2. Materials and methods

All modeling and simulation were done in parallel with custom written Python 3.8 (Python Programming Language; RRID:SCR_008394) software, run on a Cent-OS 7 server with an Intel(R) Xeon (R) E5-2630 v2 CPU.

### 2.1. Different neuron models

**Figure 1:**
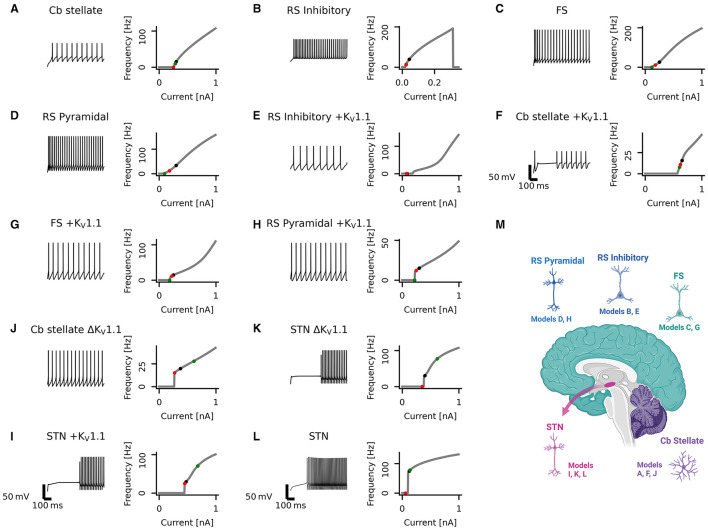
Diversity in neuronal model firing. Spike trains (left), frequency-current (fI) curves (right) for Cb stellate **(A)**, RS inhibitory **(B)**, FS **(C)**, RS pyramidal **(D)**, RS inhibitory +K_V_1.1 **(E)**, Cb stellate +K_V_1.1 **(F)**, FS +K_V_1.1 **(G)**, RS pyramidal +K_V_1.1 **(H)**, STN +K_V_1.1 **(I)**, Cb stellate ΔK_V_1.1 **(J)**, STN ΔK_V_1.1 **(K)**, and STN **(L)** neuron models. Models are sorted qualitatively based on their fI curves. Black markers on the fI curves indicate the current step at which the spike train occurs. The green marker indicates the current at which firing begins in response to an ascending current ramp, whereas the red marker indicates the current at which firing ceases in response to a descending current ramp (see Supplementary Figure 1). A schematic illustrating the anatomical locations of the models is included **(M)**; however, single-compartment models are used for each cell type.

**Figure 2:**
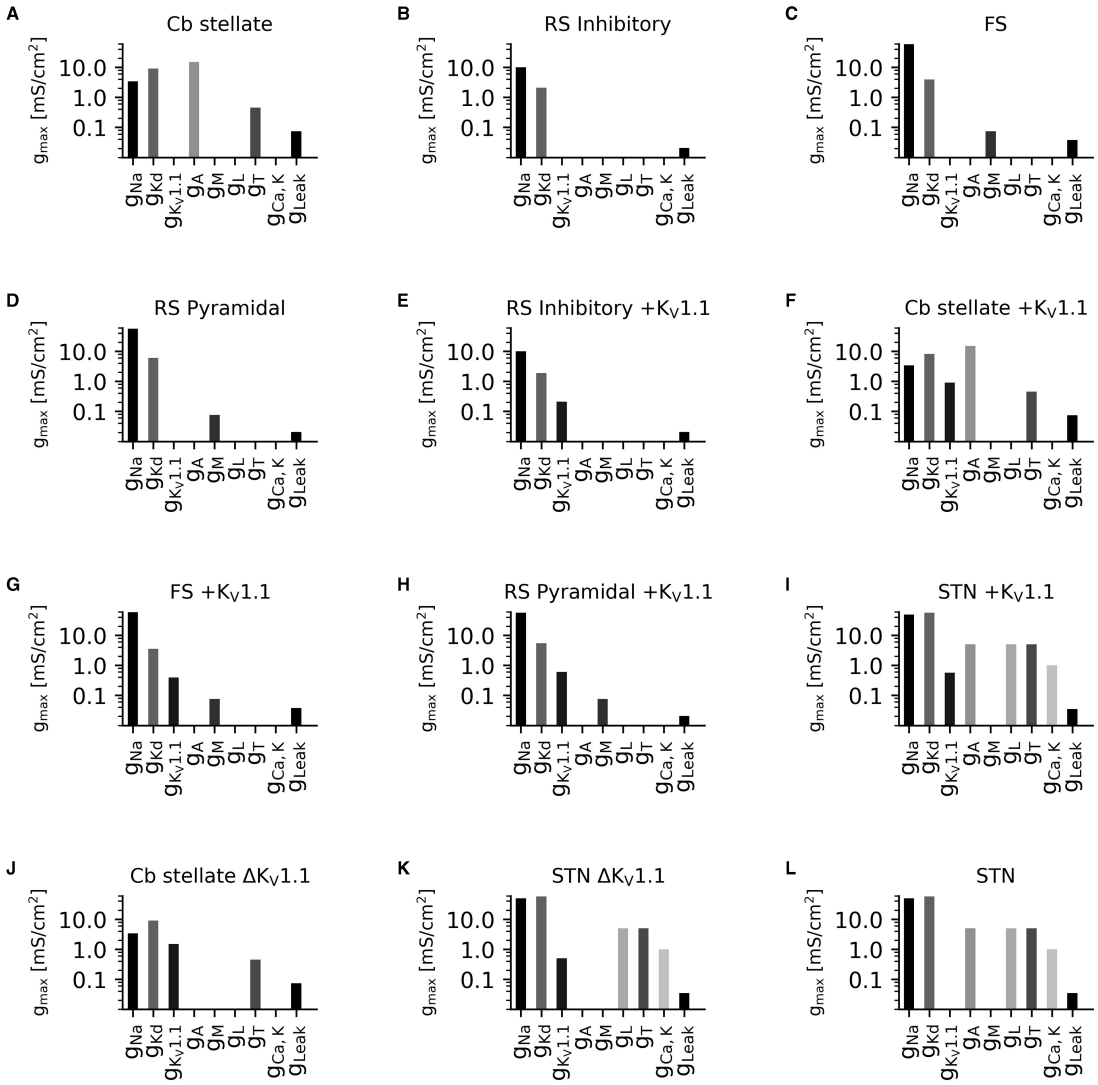
Diversity in neuronal model current composition. Distributions of maximal current conductances (g_max_) for Cb stellate **(A)**, RS inhibitory **(B)**, FS **(C)**, RS pyramidal **(D)**, RS inhibitory +K_V_1.1 **(E)**, Cb stellate +K_V_1.1 **(F)**, FS +K_V_1.1 **(G)**, RS pyramidal +K_V_1.1 **(H)**, STN +K_V_1.1 **(I)**, Cb stellate ΔK_V_1.1 **(J)**, STN ΔK_V_1.1 **(K)**, and STN **(L)** neuron models. Models are sorted as in Figure 1.

A set of single-compartment, conductance-based neuronal models representing the major classes of cortical and thalamic neurons including regular spiking pyramidal (RS pyramidal; model D), regular spiking inhibitory (RS inhibitory; model B), and fast spiking (FS; model C) neurons were used (4). Additionally, a K_V_1.1 current [I_K_V_1.1_; (43)] was added to each of these models (RS pyramidal +K_V_1.1; model H, RS inhibitory +K_V_1.1; model E, and FS +K_V_1.1; model G, respectively). A cerebellar stellate cell model from (44) is used (Cb stellate; model A) in this study. This neuron model was also extended by a K_V_1.1 current (43) either in addition to the A-type potassium current (Cb stellate +K_V_1.1; model F) or by replacing the A-type potassium current (Cb stellate ΔK_V_1.1; model J). A subthalamic nucleus (STN; model L) neuron model as described by (45) was also used. The STN neuron model (model L) was additionally extended by K_V_1.1 current (43) either in addition to the A-type potassium current (STN +K_V_1.1; model I) or by replacing the A-type potassium current (STN ΔK_V_1.1; model K). Model letter naming corresponds to panel lettering in Figure 1. The anatomical origin of each model is shown in Figure 1M. The properties and maximal conductances of each model are detailed in Table 1 and depicted in Figure 2. The gating properties are unaltered from the original Cb stellate (model A) and STN (model L) models (44, 45). For enabling the comparison of models with the typically reported electrophysiological data fitting reported and for the ease of further gating curve manipulations, a modified Boltzmann function was formed as follows:


(1) $$\begin{array}{l} x_{\infty }={\left(\frac{1-a}{1+\exp\left[\frac{V-V_{1/2}}{k}\right]}+a\right)}^{j}, \end{array}$$

with slope *k*, voltage for half-maximal activation or inactivation (*V*_1/2_), exponent *j*, and persistent current 0 ≤ *a* ≤ 1 fitted to the original formulism for RS pyramidal (model D), RS inhibitory (model B), and FS (model C) models from (4). The properties of I_K_V_1.1_ were fitted to the mean wild-type biophysical parameters of K_V_1.1 described in (39). The fitted gating parameters are detailed in Table 2. Each of the original single-compartment models used here can reproduce physiological firing behavior of the neurons they represent [Figure 1; (4, 44, 45)] and capture key aspects of the dynamics of these neuron types.

**Table 1:** Cell properties and conductances of regular spiking pyramidal neuron (RS Pyramidal; model D), regular spiking inhibitory neuron (RS inhibitory; model B), fast spiking neuron (FS; model C) each with additional I_K_V_1.1_ (RS Pyramidal +K_V_1.1; model H, RS inhibitory +K_V_1.1; model E, FS +K_V_1.1; model G, respectively), cerebellar stellate cell (Cb stellate; model A), with additional I_K_V_1.1_ (Cb stellate +K_V_1.1; model F) and with I_K_V_1.1_ replacement of I_A_ (Cb stellate ΔK_V_1.1; model J), and subthalamic nucleus neuron (STN; model L), with additional I_K_V_1.1_ (STN +K_V_1.1; model I) and with I_K_V_1.1_ replacement of I_A_ (STN K_V_1.1; model K) models.

**Model**	**RS Pyra-midal (+K_V_1.1)**	**RS inhib-itory(+K_V_1.1)**	**FS(+K_V_1.1)**	**Cbstellate**	**Cbstellate+K_V_1.1**	**CbstellateΔK_V_1.1**	**STN**	**STN+K_V_1.1**	**STNΔK_V_1.1**
	**D (H)**	**B (E)**	**C (G)**	**A**	**F**	**J**	**L**	**I**	**K**
*g*_*Na*_	56	10	58	3.4	3.4	3.4	49	49	49
*g*_*Kd*_	6 (5.4)	2.1 (1.89)	3.9 (3.51)	9.0556	8.15	9.0556	57	56.43	57
*g*_*K*_*V*_1.1_	— (0.6)	— (0.21)	— (0.39)	—	0.90556	1.50159	—	0.57	0.5
*g*_*A*_	—	—	—	15.0159	15.0159	—	5	5	—
*g*_*M*_	0.075	0.0098	0.075	—	—	—	—	—	—
*g*_*L*_	—	—	—	—	—	—	5	5	5
*g*_*T*_	—	—	—	0.45045	0.45045	0.45045	5	5	5
*g*_*Ca, K*_	—	—	—	—	—	—	1	1	1
*g*_*Leak*_	0.0205	0.0205	0.038	0.07407	0.07407	0.07407	0.035	0.035	0.035
τ_*max, M*_	608	934	502	—	—	—	—	—	—
*C*_*m*_	118.44	119.99	101.71	177.83	177.83	177.83	118.44	118.44	118.44

Capacitances (*C*_*m*_) and τ_*max, M*_ are given in pF and ms, respectively. All conductances are given in mS/cm^2^.

**Table 2:** For comparability to typical electrophysiological data fitting reported and for ease of further gating curve manipulations, a sigmoid function (Equation 1) with slope *k*, voltage for half-maximal activation or inactivation (*V*_1/2_), exponent *j*, and persistent current 0 ≤ *a* ≤ 1 were fitted for the models originating from Pospischil et al. (4) (models B, C, D, E, G, H) where α_*x*_ and β_*x*_ are used.

	**Gating**	***V*_1/2_ [mV]**	***k***	***j***	***a***
Models	I_Na_ activation	−34.33054521	−8.21450277	1.42295686	—
B, C, D, E, G, H	I_Na_ inactivation	−34.51951036	4.04059373	1	0.05
	I_Kd_ activation	−63.76096946	−13.83488194	7.35347425	—
	I_L_ activation	−39.03684525	−5.57756176	2.25190197	—
	I_L_ inactivation	−57.37	20.98	1	—
	I_M_ activation	−45	−9.9998807337	1	—
I_K_V_1.1_	I_K_V_1.1_ activation	−30.01851852	−7.73333333	1	—
	I_K_V_1.1_ Inactivation	−46.85851852	7.67266667	1	0.245

Gating parameters for I_K_V_1.1_ are taken from Ranjan et al. (43) and fit to mean wild type parameters in Lauxmann et al. (39). Model gating parameters not listed are taken directly from source publication.

### 2.2. Firing frequency analysis

The membrane responses to 200 equidistant 2 s long current steps were simulated using the forward Euler method with a Δt = 0.01 ms from a steady state. Current steps ranged from 0 to 1 nA (step size 5 pA) for all models except for the RS inhibitory neuron models, where a range of 0–0.35 nA (step size 1.75 pA) was used to ensure repetitive firing across the range of input currents. For each current step, action potentials were detected as peaks with at least 50 mV prominence, or the relative height above the lowest contour line encircling it, and a minimum interspike interval of 1 ms. The interspike interval was computed and used to determine the instantaneous firing frequencies elicited by the current step. A ramp protocol, consisting of a 2 s ascending ramp followed by a 2 s descending ramp, was also simulated over the same current range to assess model hysteresis. Rheobases assessed from this ramp protocol were not used for subsequent analysis in order to maintain relatability to commonly used experimental measures.

**Figure 3:**
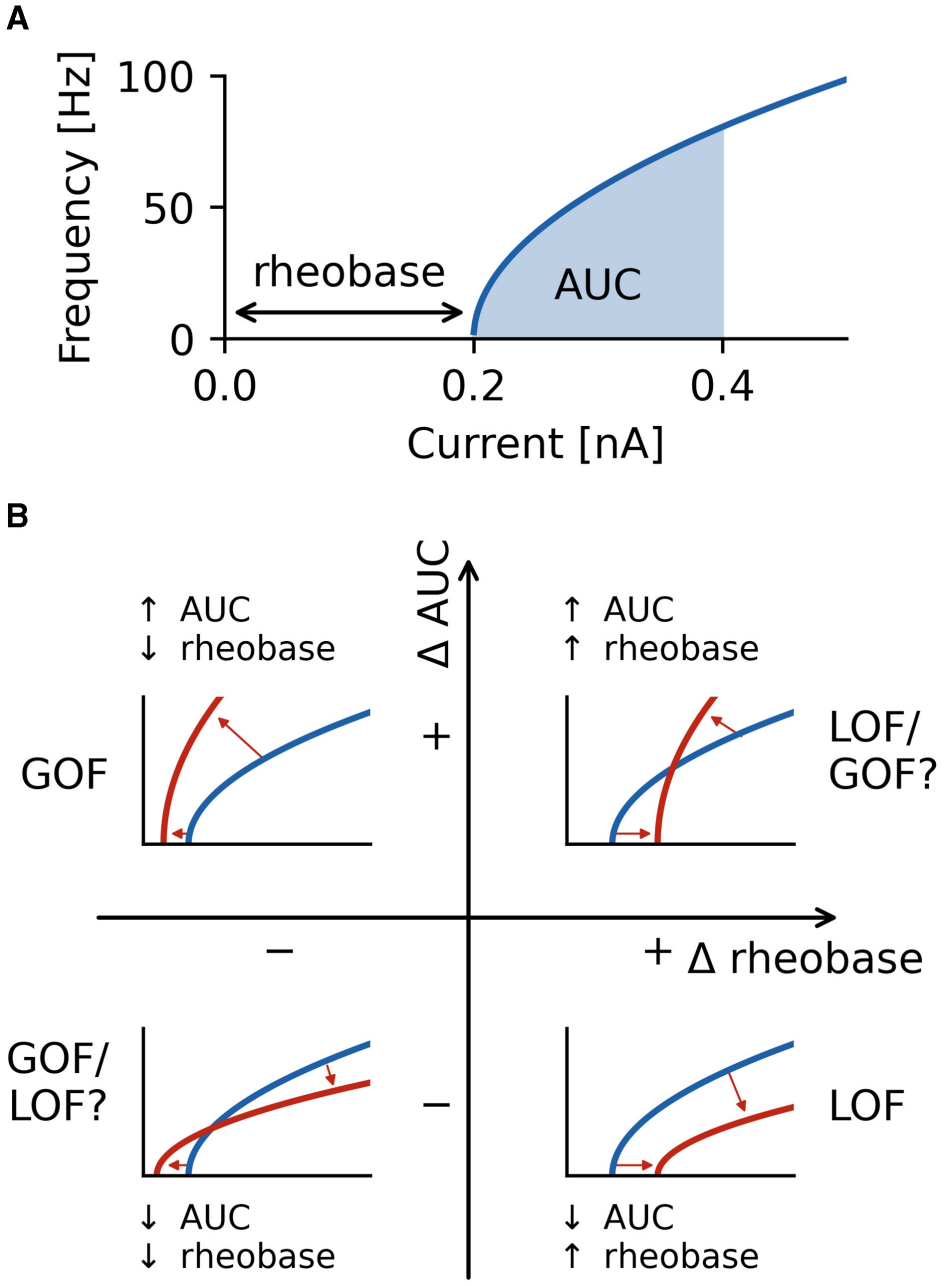
Characterization of firing with AUC and rheobase. **(A)** The area under the curve (AUC) of the repetitive firing frequency-current (fI) curve. **(B)** Changes in firing as characterized by ΔAUC and Δrheobase occupy four quadrants separated by no changes in AUC and rheobase. Representative schematic fI curves in red with respect to a reference (or wild type) fI curve (blue) depict the general changes associated with each quadrant. Square root functions are used as fI curves for illustration purposes.

To ensure accurate firing frequencies at low firing rates and reduced spike sampling bias, steady-state firing was defined as the mean firing frequency in a 500 ms window in the last second of the current steps starting at the initial action potential in this last second. Firing characterization was performed in the last second of current steps to ensure steady-state firing is captured, and adaptation processes are neglected in our analysis. Alteration in current magnitudes can have different effects on rheobase and the initial slope of the fI curve (30). For this reason, we quantified neuronal firing using the rheobase as well as the area under the curve (AUC) of the initial portion of the fI curve as a measure of the initial slope of the fI curve (Figure 3A).

The smallest current at which steady state firing occurred was identified, and the current step interval preceding the occurrence of steady state firing was simulated at higher resolution (100 current steps) to determine the current at which steady state firing began. Firing was simulated with 100 current steps from this current upwards for 1/5 of the overall current range. Over this range, a fI curve was constructed and the integral, or area under the curve (AUC), of the fI curve over this interval was computed with the composite trapezoidal rule and used as a measure of firing rate independent from rheobase.

To obtain the rheobase at a higher current resolution than the fI curve, the current step interval preceding the occurrence of action potentials was explored at higher resolution with 100 current steps spanning the interval (step sizes of 0.05 and 0.0175 pA, respectively). Membrane responses to these current steps were then analyzed for action potentials, and the rheobase was considered the lowest current step for which an action potential was elicited.

All models exhibited tonic steady-state firing with default parameters. In limited instances, variations of parameters elicited periodic bursting; however, these instances were excluded from further analysis.

### 2.3. Sensitivity analysis and comparison of models

Properties of ionic currents common to all models (I_Na_, I_Kd_, I_A_/I_K_V_1.1_, and I_Leak_) were systematically altered in a one-factor-at-a-time sensitivity analysis for all models. The gating curves for each current were shifted (Δ*V*_1/2_) from −10 to 10 mV with an increment of 1 mV. The voltage dependence of the time constant associated with the shifted gating curve was correspondingly shifted. The slope (*k*) of the gating curves were altered from half to twice the initial slope. Similarly, the maximal current conductance (*g*) was also scaled from half to twice the initial value. For both slope and conductance alterations, alterations consisted of 21 steps spaced equally on a log_2_ scale. We neglected the variation of time constants for the practical reason that estimation and assessment of time constants and changes to them is not straightforward (46, 47).

### 2.4. Model comparison

Changes in rheobase (Δrheobase) were calculated in relation to the original model rheobase. The contrast of each AUC value (*AUC*_*i*_) was computed in comparison to the AUC of the unaltered wild type model (*AUC*_*wt*_):


(2) $$\begin{array}{c} \text{normalized}\Delta \text{AUC}=\frac{AUC_{i}-AUC_{wt}}{AUC_{wt}}. \end{array}$$

To assess whether the effects of a given alteration on normalized ΔAUC or Δrheobase were robust across models, the correlation between normalized ΔAUC or Δrheobase and the magnitude of the alteration of a current property was computed for each alteration in each model and compared across alteration types. The Kendall's τ coefficient, a non-parametric rank correlation, is used to describe the relationship between the magnitude of the alteration and AUC or rheobase values. A Kendall τ value of −1 or 1 is indicative of monotonically decreasing and increasing relationships, respectively.

### 2.5. *KCNA1* mutations

Known episodic ataxia type 1 associated *KCNA1* mutations and their electrophysiological characterization have been reviewed in (39). The mutation-induced changes in I_K_V_1.1_ amplitude and activation slope (*k*) were normalized to wild-type measurements, and changes in activation *V*_1/2_ were used relative to wild-type measurements. Although initially described as having a lack of fast activation, K_V_1.1 displays prominent inactivation at physiologically relevant temperatures (43). The effects of a mutation were also applied to I_A_ when present as both potassium currents display inactivation. In all cases, the mutation effects were applied to half of the K_V_1.1 or I_A_ under the assumption that the heterozygous mutation results in 50% of channels carrying the mutation. Frequency-current curves for each mutation in each model were obtained through simulation and used to characterize firing behavior as described above. For each model, the differences in mutation AUC to wild type AUC were normalized by wild-type AUC (normalized ΔAUC) and mutation rheobases were compared to wild-type rheobase values (Δrheobase). Pairwise Kendall rank correlations (Kendall τ) were used to compare the correlation in the effects of K_V_1.1 mutations on AUC and rheobase between models.

### 2.6. Code accessibility

The simulation and analysis code including full specification of the models is freely available online at https://github.com/nkoch1/LOFGOF2023.

## 3. Results

To examine the role of neuron-type specific ionic current environments on the impact of altered ion currents properties on firing behavior, we performed those as follows: (1) firing responses were characterized with rheobase and ΔAUC, (2) a set of neuronal models was used, and properties of channels common across models were altered systematically one at a time, and (3) the effects of a set of episodic ataxia type 1 associated *KCNA1* mutations on firing was then examined across different neuronal models with different ionic current environments.

### 3.1. Variety of model neurons

Neuronal firing is heterogeneous across the CNS, and a set of neuronal models with heterogeneous firing due to different ionic currents is desirable to reflect this heterogeneity. The set of single-compartment, conductance-based neuronal models used here has considerable diversity, as evident in the variability seen across neuronal models both in spike trains and their fI curves (Figure 1). The models chosen for this study all fire tonically and do not exhibit bursting (see Section 2 for details and naming of the models). Models are qualitatively sorted based on their firing curves and labeled model A through L accordingly. Model B ceases firing with large current steps (Figure 1B) indicating depolarization block. Some models, such as models A and B, display type I firing, whereas others such as models J and L exhibit type II firing. Type I firing is characterized by continuous fI curves (i.e., firing rate increases from 0 in a continuous fashion) whereas type II firing is characterized by a discontinuity in the fI curve [i.e., a jump occurs from no firing to firing at a certain frequency; (48, 49)]. The other models used here lie on a continuum between these prototypical firing classifications. Most neuronal models exhibit hysteresis with ascending and descending ramps eliciting spikes at different current thresholds. However, the models I, J, and K have large hysteresis (Figure 1 and Supplementary Figure 1). Different types of underlying current dynamics are known to generate these different firing types and hysteresis (48, 50, 51). This broad range of single-compartmental models represents the distinct dynamics of various neuron types across diverse brain regions but does not take into account differences in morphology or synaptic input.

### 3.2. Characterization of neuronal firing properties

Neuronal firing is a complex phenomenon, and a quantification of firing properties is required for comparisons across neuron types and between different conditions. In this study, we focus on two aspects of firing that are routinely measured in clinical settings (52): rheobase, the smallest injected current at which the neuron fires an action potential, and the shape of the frequency-current (fI) curve as quantified by the area under the curve (AUC) for a fixed range of input currents above rheobase (Figure 3A). The characterization of the firing properties of a neuron by using rheobase and AUC allows to characterize both a neuron's excitability in the sub-threshold regime (rheobase) and periodic firing in the super-threshold regime (AUC) by two independent measures. Note that AUC is essentially quantifying the slope of a neuron's fI curve.

Using these two measures, we quantified the effects a changed property of an ionic current has on neural firing by the differences in both rheobase, Δrheobase, and in AUC, ΔAUC, relative to the wild type neuron. ΔAUC is in addition normalized to the AUC of the wild type neuron, see Equation (2). Each fI curve resulting from an altered ionic current is a point in a two-dimensional coordinate system spanned by Δrheobase and normalized ΔAUC (Figure 3B). An fI curve similar to the one of the wild type neuron is marked by a point close to the origin. In the upper left quadrant, fI curves become steeper (positive difference of AUC values: +ΔAUC) and are shifted to lower rheobases (negative difference of rheobases: −Δrheobase), unambiguously indicating an increased firing that clearly might be classified as a gain of function (GOF) of neuronal firing. The opposite happens in the bottom right quadrant where the slope of fI curves decreases (−ΔAUC), and the rheobase is shifted to higher currents (+Δrheobase), indicating a decreased, loss of function (LOF) firing. In the lower left (−ΔAUC and −Δrheobase) and upper right (+ΔAUC and +Δrheobase) quadrants, the effects on firing are less clear-cut because the changes in rheobase and AUC have opposite effects on neuronal firing. Changes in a neuron's fI curves in these two quadrants cannot uniquely be described as a gain or loss of excitability.

### 3.3. Sensitivity analysis

Sensitivity analyses are used to understand how input model parameters contribute to determining the output of a model (53). In other words, sensitivity analyses are used to understand how sensitive the output of a model is to a change in input or model parameters. One-factor-a-time sensitivity analyses involve altering one parameter at a time and assessing the impact of this parameter on the output. This approach enables the comparison of given alterations in parameters of ionic currents across models.

**Figure 4:**
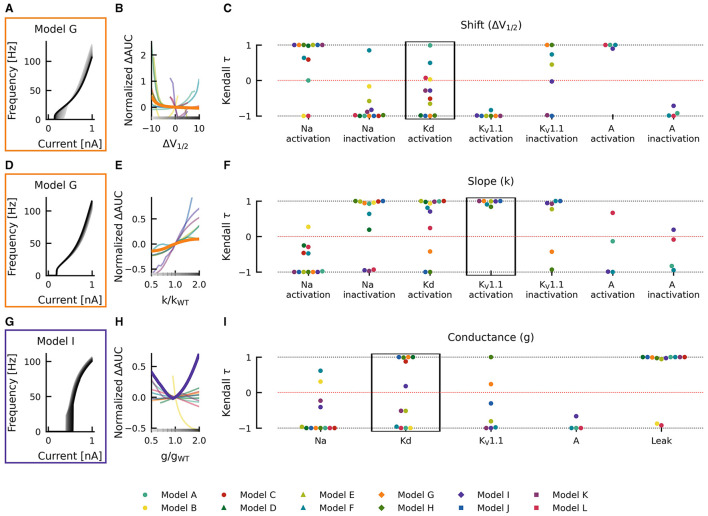
Effects of altered channel kinetics on AUC in various neuron models. The fI curves corresponding to shifts in model G delayed rectifier Kd half activation *V*_1/2_ **(A)**, changes K_V_1.1 activation slope factor *k* in model G **(D)**, and changes in maximal conductance of delayed rectifier K current in the model I **(G)** are shown. The fI curves from the smallest (gray) to the largest (black) alterations are seen for **(A, D, G)** in accordance to the grayscale of the x-axis in **(B, E, H)**. The normalized ΔAUC of fI curves is plotted against delayed rectifier K half activation potential [Δ*V*_1/2_; **(B)**], K_V_1.1 activation slope factor *k* [k/k_WT_; **(E)**], and maximal conductance *g* of the delayed rectifier Kd current [g/g_WT_; **(H)**] for all models (thin lines) with relationships from the fI curve examples [**(A, D, G)**, respectively] highlighted by thick lines with colors corresponding to the box highlighting each set of fI curves. The Kendall rank correlation (Kendall τ) coefficients between shifts in half maximal potential *V*_1/2_ and normalized ΔAUC **(C)**, slope factor k and normalized ΔAUC **(F)**, as well as maximal current conductances and normalized ΔAUC **(I)** for each model and current property is computed. The relationships between Δ*V*_1/2_, k/k_WT_, and g/g_WT_ and normalized ΔAUC for the Kendall rank correlations highlighted in the black boxes are depicted in **(B, E, H)**, respectively.

For example, when shifting the half activation voltage *V*_1/2_ of the delayed rectifier potassium current in the model G to more depolarized values, then the rheobase of the resulting fI curves shifted to lower currents −Δrheobase, making the neuron more sensitive to weak inputs, but at the same time, the slope of the fI curves was reduced (−normalized ΔAUC), which resulted in a reduced firing rate (Figure 4A). As a result, the effect of a depolarizing shift in the delayed rectifier potassium current half activation *V*_1/2_ in model G is in the bottom left quadrant of Figure 4B and characterization as LOF or GOF in excitability is not possible. Plotting the corresponding changes in AUC against the change in half activation potential *V*_1/2_ results in a monotonically falling curve (thick orange line in Figure 4B). For each of the many models, we got a different relation between the changes in AUC and the shifts in half maximal potential *V*_1/2_ (thin lines in Figure 4B). To further summarize these different dependencies of the various models, we characterized each of these curves by a single number, the Kendall τ correlation coefficient, a monotonically increasing curve resulted in a Kendall τ close to +1 a monotonously decreasing curve in Kendall τ≈−1, and a non-monotonous, non-linear relation in Kendall τ close to zero (compare lines in Figure 4B with dots in black box in Figure 4C).

Changes in gating half activation potential *V*_1/2_ and slope factor *k* as well as the maximum conductance *g* affected the AUC (Figure 4), but how exactly the AUC was affected usually depended on the specific neuronal model. Increasing the slope factor of the K_V_1.1 activation curve, for example, increased the AUC in all models (Kendall τ≈+1) but with different slopes (Figures 4D–F). Similar consistent positive correlations could be found for shifts in A-current activation *V*_1/2_. Changes in K_V_1.1 half activation *V*_1/2_ and in maximal A-current conductance resulted in negative correlations with the AUC in all models (Kendall τ≈−1).

Qualitative differences could be found, for example, when increasing the maximal conductance of the delayed rectifier (Figures 4G–I). In some model neurons, this increased AUC (Kendall τ≈+1), whereas in others, AUC was decreased (Kendall τ≈−1). In model I, AUC depended in a non-linear way on the maximal conductance of the delayed rectifier, resulting in a Kendall τ close to zero. Even more dramatic qualitative differences between models resulted from shifts of the activation curve of the delayed rectifier, as discussed already above (Figures 4A–C). Some model neurons did almost not depend on changes in Kd-current half activation *V*_1/2_ or showed strong non-linear dependencies, both resulting in Kendall τ close to zero. Many model neurons showed strongly negative correlations, and a few displayed positive correlations with shifting the activation curve of the delayed rectifier.

**Figure 5:**
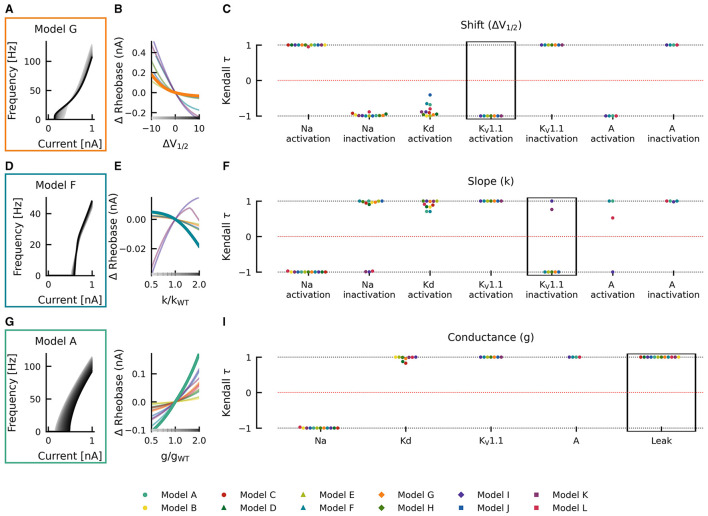
Effects of altered channel kinetics on rheobase. The fI curves corresponding to shifts in model G K_V_1.1 activation *V*_1/2_ **(A)**, changes K_V_1.1 inactivation slope factor *k* in model F **(D)**, and changes in maximal conductance of the leak current in model A **(G)** are shown. The fI curves from the smallest (gray) to the largest (black) alterations are seen for **(A, D, G)** in accordance to the grayscale of the x-axis in **(B, E, H)**. The Δrheobase of fI curves is plotted against K_V_1.1 half activation potential [Δ*V*_1/2_; **(B)**], K_V_1.1 inactivation slope factor *k* [k/k_WT_; **(E)**], and maximal conductance *g* of the leak current [g/g_WT_; **(H)**] for all models (thin lines) with relationships from the fI curve examples [**(A, D, G)**, respectively] highlighted by thick lines with colors corresponding to the box highlighting each set of fI curves. The Kendall rank correlation (Kendall τ) coefficients between shifts in half maximal potential *V*_1/2_ and Δrheobase **(C)**, slope factor k and Δrheobase **(F)** as well as maximal current conductances and Δrheobase **(I)** for each model and current property is computed. The relationships between Δ*V*_1/2_, k/k_WT_, and g/g_WT_ and Δrheobase for the Kendall rank correlations highlighted in the black boxes are depicted in **(B, E, H)**, respectively.

Changes in gating half activation potential *V*_1/2_ and slope factor *k* as well as the maximum conductance *g* affected rheobase (Figure 5). However, in contrast to AUC, qualitatively consistent effects on rheobase across models could be observed. An increase in the maximal conductance of the leak current in model A increased the rheobase (Figure 5G). When these changes were plotted against the change in maximal conductance, a monotonically increasing relationship was evident (thick teal line in Figure 5H). This monotonically increasing relationship was evident in all models (Kendall τ≈+1) but with different slopes (thin lines in Figure 5H). Similarly, positive correlations were consistently found across models for maximal conductances of delayed rectifier Kd, K_V_1.1, and A-type currents, whereas the maximal conductance of the sodium current was consistently associated with negative correlations (Kendall τ≈−1; Figure 5I), i.e., rheobase decreased with increasing maximum conductance in all models.

Although changes in half maximal potential *V*_1/2_ and slope factor *k* are generally correlated with rheobase, similarly across models, there were some exceptions. Rheobase was affected with both, with positive and negative correlations in different models as a result of changing slope factor of Na^+^-current inactivation (positive: models A–H and J; negative: models I, K, and L), K_V_1.1-current inactivation (positive: models I and K; negative: models E–G, J, H), and A-current activation (positive: models A, F and L; negative: model I; Figure 5F). Departures from monotonic relationships also occurred in some models as a result of Kd-current activation *V*_1/2_ (e.g., model J) and slope factor *k* (models F and G), K_V_1.1-current inactivation slope factor *k* (model K), and A-current activation slope factor *k* (model L). Thus, identical changes in current gating properties such as the half maximal potential *V*_1/2_ or slope factor *k* can have differing effects on firing depending on the model in which they occur.

### 3.4. *KCNA1* mutations

**Figure 6:**
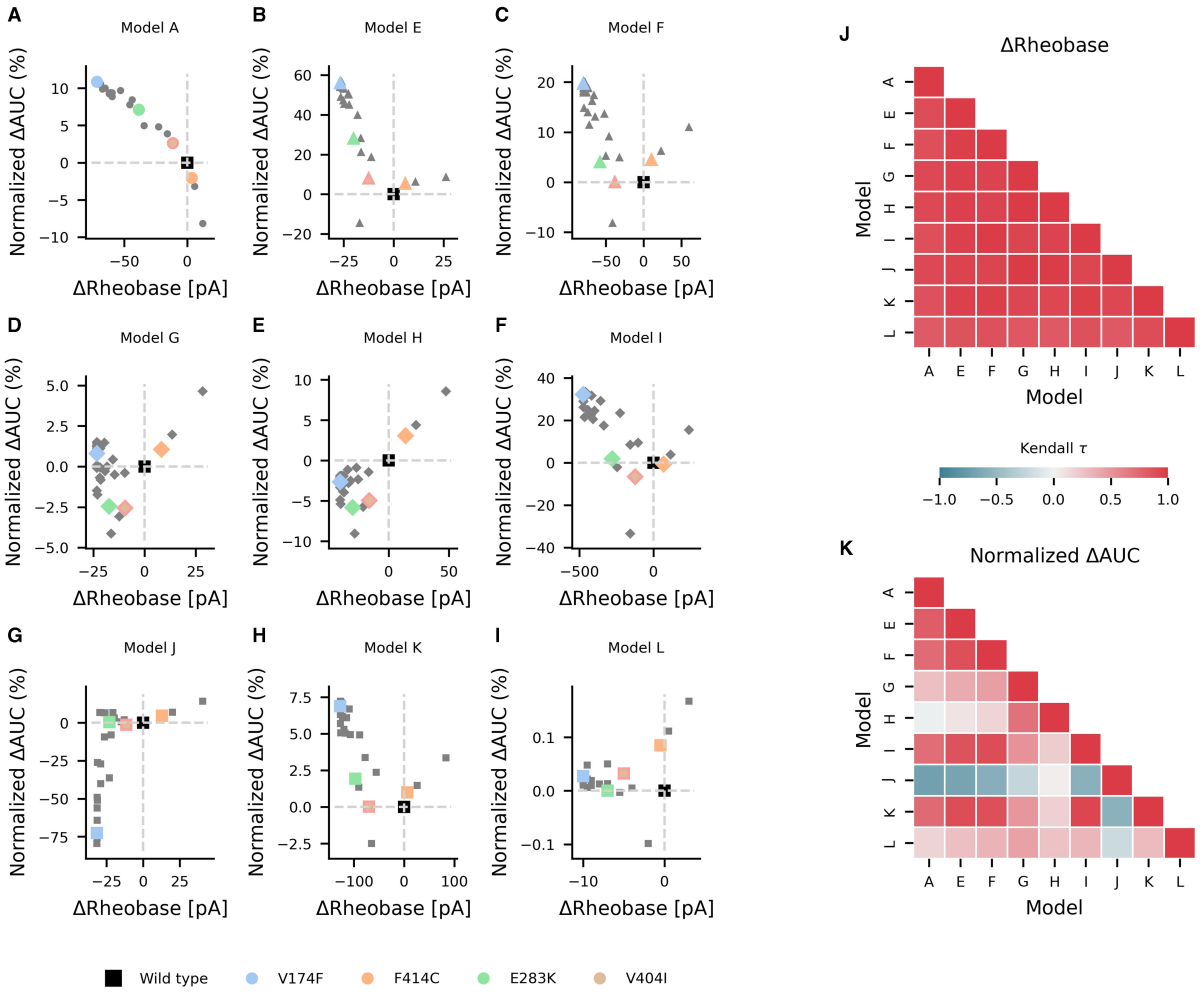
Effects of episodic ataxia type 1 associated *KCNA1* mutations on firing. Effects of *KCNA1* mutations on AUC (percent change in normalized ΔAUC) and rheobase (ΔRheobase) compared to wild type for model H **(A)**, model E **(B)**, model G **(C)**, model A **(D)**, model F **(E)**, model J **(F)**, model L **(G)**, model I **(H)**, and model K **(I)**. All *KCNA1* mutations are marked in gray with the V174F, F414C, E283K, and V404I *KCNA1* mutations highlighted in color for each model. Pairwise Kendall rank correlation coefficients (Kendall τ) between the effects of *KCNA1* mutations on rheobase and on AUC are shown in **(J, K)**, respectively. A marker shape is indicative of model/firing type, and gray dashed lines denote the quadrants of firing characterization (see Figure 3).

Mutations in *KCNA1* are associated with episodic ataxia type 1 (EA1) and have been characterized biophysically [as reviewed by (39)]. In the study, they were used as a test case in the effects of various ionic current environments on neuronal firing and on the outcomes of channelopathies. The changes in AUC and rheobase from wild type values for reported EA1 associated *KCNA1* mutations were heterogeneous across models containing K_V_1.1 but generally showed a decrease in rheobase (Figures 6A–I). Pairwise non-parametric Kendall τ rank correlations between the simulated effects of these K_V_1.1 mutations on rheobase were highly correlated across models (Figure 6J), indicating that EA1 associated *KCNA1* mutations generally decrease rheobase across diverse neuron types. However, the effects of the K_V_1.1 mutations on AUC were more heterogenous as reflected by both weak and strong positive and negative pairwise correlations between models (Figure 6K), suggesting that the effects of ion-channel variant on super-threshold neuronal firing depend both quantitatively and qualitatively on the specific composition of ionic currents in a given neuron.

## 4. Discussion

To compare the effects of ion channel mutations on neuronal firing of different neuron types, we used a diverse set of conductance-based models, that differ in their composition of ionic currents, to systematically characterize the effects of changes in individual channel properties. Additionally, we simulated the effects of specific episodic ataxia type 1 associated (EA1) *KCNA1* mutations. Changes to single ionic current properties, as well as known EA1 associated *KCNA1* mutations showed consistent effects on the rheobase across neuron types, whereas the effects on the slope of the steady-state fI-curve depended on the neuron type. Our results demonstrate that loss of function (LOF) and gain of function (GOF) on the biophysical level cannot be uniquely transferred to the level of neuronal firing. Thus, the effects caused by different mutations depend on the properties of the other ion channels expressed in a neuron and therefore depend on the channel ensemble of a specific neuron type.

### 4.1. Firing frequency analysis

Although differences in neuronal firing can be characterized by an area under the curve of the fI curve for a fixed current range, this approach characterizes firing as a mixture of two key features: rheobase and the initial slope of the fI curve. By probing rheobase directly and using an AUC relative to rheobase, we disambiguate these features and enable insights into the effects on rheobase and initial fI curve steepness. This increase in the specificity of our understanding of how ion channel mutations alter firing across neuron types and enable classification is described in Figure 3. Importantly, in cases when ion channel mutations alter rheobase and initial fI curve steepness in ways that oppose effects on firing (upper left and bottom right quadrants of Figure 3B), this disambiguation is important for understanding the outcome of the mutation. In these cases, the regime the neuron is operating in is vital in determining the neuron's firing outcome. If it is in its excitable regime and only occasionally generates an action potential, then the effect on the rheobase is more important. If it is firing periodically with high rates, then the change in AUC might be more relevant.

### 4.2. Modeling limitations

The single-compartment models used here all capture key aspects of the firing dynamics for their respective neuron. The models fall short of capturing the morphology, complex physiology, biophysics, and heterogeneity of real neurons nor do they take into account subunit stoichiometry, auxiliary subunits, or membrane composition which influence the biophysics of ionic currents (54–57). However, these simplified models allow to study the effect of different compositions of ionic currents on the diversity in firing outcomes of ion channel mutations in isolation.

Our results demonstrate that for exploring possible neuron-type specific effects, variety of currents and dynamics across models is of utmost importance. With this context in mind, the collection of models used here are labeled as models A-L to highlight that the physiological neurons they represent is not of chief concern but rather that the collection of models with different attributes respond heterogeneously to the same perturbation. Additionally, the development of more realistic models is a high priority and will enable neuron-type specific predictions that may aid precision medicine approaches. Thus, weight should not be put on any single predicted firing outcome here in a specific model but rather on the differences in outcomes that occur across the neuron-type spectrum the models used here represent. Further investigation and analysis of the neuron-type effects of ion channel mutations including animal experiments is essential for validation of the results presented here and for furthering the understanding of the effects of channelopathies at multiple levels of scale.

### 4.3. Neuronal diversity

The nervous system consists of a vastly diverse and heterogeneous collection of neurons with variable properties and characteristics including diverse combinations and expression levels of ion channels which are vital for neuronal firing dynamics.

Advances in high-throughput techniques have enabled large-scale investigation into single-cell properties across the CNS (58) that have revealed large diversity in neuronal gene expression, morphology and neuronal types in the motor cortex (28), neocortex (26, 59), GABAergic neurons in the cortex and retina (60, 61), cerebellum (62), spinal cord (63), visual cortex (64) as well as the retina (65–69).

Diversity across neurons is not limited to gene expression and can also be seen electrophysiologically (28, 59, 64, 65, 67, 70–72) with correlations existing between gene expression and electrophysiological properties (70). At the ion channel level, diversity exists not only between the specific ion channels the different neuron types express but heterogeneity also exists in ion channel expression levels within neuron types (32, 73, 74). As ion channel properties and expression levels are key determinants of neuronal dynamics and firing (30, 75–82) neurons with different ion channel properties and expression levels display different firing properties.

To capture the diversity in neuronal ion channel expression and its relevance in the outcome of ion channel mutations, we used multiple neuronal models with different ionic currents (Figure 2) and underlying firing dynamics (Figure 3).

### 4.4. Ionic current environments determine the effect of ion channel mutations

To the best of our knowledge, no comprehensive evaluation of how ionic current environment and neuron type affect the outcome of ion channel mutations has been reported. However, comparisons between the effects of such mutations between certain neuron types were described. For instance, the R1648H mutation in *SCN1A* does not alter the excitability of cortical pyramidal neurons but causes hypoexcitability of adjacent inhibitory GABAergic neurons (24). In the CA3 region of the hippocampus, the equivalent mutation in *SCN8A*, R1627H, increases the excitability of pyramidal neurons and decreases the excitability of parvalbumin positive interneurons (33). Additionally, the L858H mutation in Na_V_1.7, associated with erythromelalgia, has been shown to cause hypoexcitability in sympathetic ganglion neurons and hyperexcitability in dorsal root ganglion neurons (83, 84). The differential effects of L858H Na_V_1.7 on firing is dependent on the presence or absence of another sodium channel, namely, the Na_V_1.8 subunit (83, 84). These findings, in concert with our findings emphasize that the ionic current environment in which a channelopathy occurs is vital in determining the outcomes of the channelopathy on firing. However, many differences can exist between neuron types at multiple levels of the scale not only in ion channel composition. Despite this complexity, the simulations performed here demonstrate that differential ion channel composition is sufficient to cause neuron-type differences in the effects of ion channel mutations.

Neuron-type specific differences in ionic current properties are important in the effects of ion channel mutations. However, within a neuron-type heterogeneity, in channel expression levels exists, and it is often desirable to generate a population of neuronal models and to screen them for plausibility to biological data in order to capture neuronal population diversity (73, 85). The models we used here are originally generated by characterization of current gating properties and by fitting of maximal conductances to experimental data (4, 43–45). This practice of fixing maximal conductances based on experimental data is limiting as it does not reproduce the variability in channel expression and neuronal firing behavior of a heterogeneous neuron population (86). For example, a model derived from the mean conductance in a neuronal sub-population within the stomatogastric ganglion, the so-called “one-spike bursting” neurons fire three spikes instead of one per burst due to an L-shaped distribution of sodium and potassium conductances (31). Multiple sets of conductances can give rise to the same patterns of activity also termed degeneracy, and differences in neuronal dynamics may only be evident with perturbations (73, 74). The variability in ion channel expression often correlates with the expression of other ion channels (74) and neurons whose behavior is similar may possess correlated variability across different ion channels resulting in stability in the neuronal phenotype (87–89). The variability of ionic currents and degeneracy of neurons may account, at least in part, for the observation that the effect of toxins within a neuronal type is frequently not constant (90–92).

### 4.5. Effects of *KCNA1* mutations

Changes in delayed rectifier potassium currents, analogous to those seen in LOF *KCNA1* mutations, change the underlying firing dynamics of the Hodgkin-Huxley model that result in reduced thresholds for repetitive firing and thus contribute to increased excitability (93). Although the Hodgkin-Huxley delayed rectifier lack inactivation, the increase in excitability observed by (93) is in line with our simulation-based predictions of the outcomes of *KCNA1* mutations. LOF *KCNA1* mutations generally increase neuronal excitability; however, the varying susceptibility on rheobase and different effects on AUC of the fI-curve of *KCNA1* mutations across models are indicative that a certain neuron type specific complexity exists. Increased excitability is seen experimentally with K_V_1.1 null mice (94, 95), with pharmacological K_V_1.1 block (96, 97) and by (93) with simulation-based predictions of *KCNA1* mutations. Contrary to these results, Zhao et al. (98) predicted *in silico* that the depolarizing shifts seen as a result of *KCNA1* mutations broaden action potentials and interfere negatively with high frequency action potential firing. However, they varied stimulus duration between different models and therefore comparability of firing rates is lacking in this study.

In our simulations, different current properties alter the impact of *KCNA1* mutations on firing as evident in the differences seen in the impact of I_A_ and I_K_V_1.1_ in the Cb stellate and STN model families on *KCNA1* mutation firing. This highlights that not only knowledge of the biophysical properties of a channel but also its neuronal expression and other neuronal channels present is vital for the holistic understanding of the effects of a given ion channel mutation both at the single cell and network level.

### 4.6. Loss or gain of function characterizations do not fully capture ion channel mutation effects on firing

The effects of changes in channel properties depend in part on the neuronal model in which they occur and can be seen in the variance of correlations (especially in AUC of the fI-curve) across models for a given current property change. Therefore, relative conductances and gating properties of currents in the ionic current environment in which an alteration in current properties occurs play an important role in determining the outcome on firing. The use of LOF and GOF is useful at the level of ion channels to indicate whether a mutation results in more or less ionic current. However, the extension of this thinking onto whether mutations induce LOF or GOF at the level of neuronal firing based on the ionic current LOF/GOF is problematic because of this dependency of neuronal firing changes on the ionic channel environment. Thus, the direct leap from current level LOF/GOF characterizations to effects on firing without experimental or modeling-based evidence, although tempting, should be refrained from and viewed with caution when reported. This is especially relevant in the recent development of personalized medicine for channelopathies, where a patient's specific channelopathy is identified and used to tailor treatments (5, 99–104). In these cases, the effects of specific ion channel mutations are often characterized based on ionic currents in expression systems and classified as LOF or GOF to aid in treatment decisions (5, 38, 103). Although positive treatment outcomes occur with sodium channel blockers in patients with GOF Na_V_1.6 mutations, patients with both LOF and GOF Na_V_1.6 mutations can benefit from treatment with sodium channel blockers (38). This example suggests that the relationship between effects at the level of ion channels and effects at the level of firing and therapeutics is not linear or evident without further contextual information.

Therefore, the transfer of LOF or GOF from the current to the firing level should be used with caution; the neuron type in which the mutant ion channel is expressed may provide valuable insight into the functional consequences of an ion channel mutation. Experimental assessment of the effects of a patient's specific ion channel mutation *in vivo* is not generally feasible at a large scale. Therefore, modeling approaches investigating the effects of patient specific channelopathies provide a viable method bridging between characterization of changes in biophysical properties of ionic currents and the firing consequences of these effects. In both experimental and modeling studies on the effects of ion channel mutations on neuronal firing, the specific dependency on neuron type should be considered.

**Figure 7:**
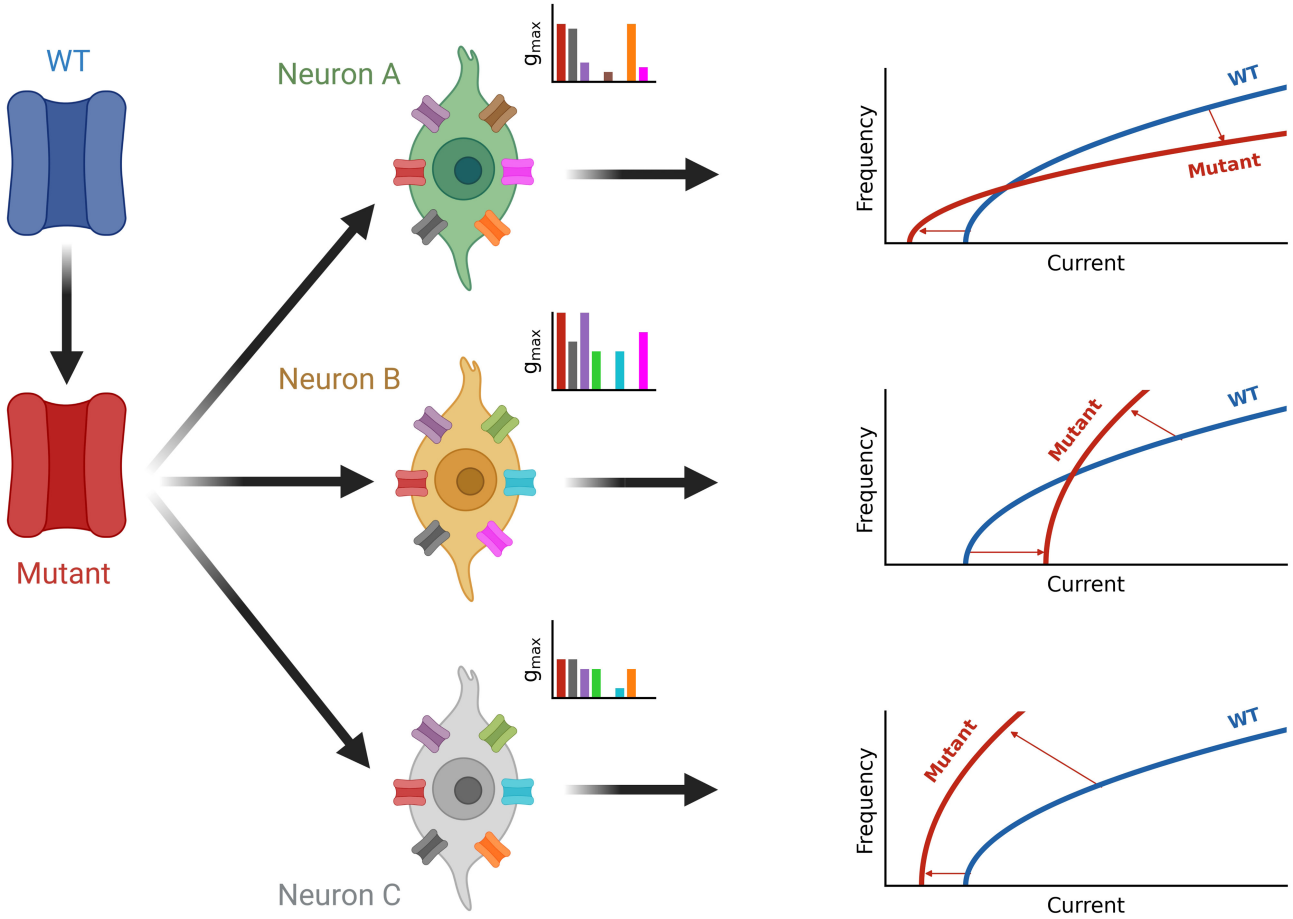
Summary of neuron-type dependence of channelopathies. A wild-type channel (WT, blue) is mutated (Mutant, red) and expressed in different neuron types (green, orange, and gray) each with a unique set of ion channels (see inset axes). The current composition of each neuron determines the effect of firing seen by the shift from the blue wild type fI curve to the red fI curve for the mutated ion channel on the right. Square root functions are used as fI curves for illustration purposes.

Our simulations demonstrate that the effects of altered ion channel properties on firing is generally influenced by the other ionic currents present in the neuron as illustrated in Figure 7. In channelopathies, the effect of a given ion channel mutation on neuronal firing therefore depends on the neuron type in which those changes occur (24, 33, 83, 84). Although certain complexities of neurons such as differences in neuron-type sensitivities to current property changes, interactions between ionic currents, cell morphology, and subcellular ion channel distribution are neglected here, and it is likely that this increased complexity *in vivo* would contribute to the neuron-type dependent effects on neuronal firing. The complexity and nuances of the nervous system, including neuron-type dependent firing effects of channelopathies explored here, likely underlie shortcomings in treatment approaches in patients with channelopathies. Accounting for neuron-type dependent firing effects provides an opportunity to improve the efficacy and precision in personalized medicine approaches. Although this is not experimentally feasible, improved modeling and simulation methods to predict neuron-type dependent effects may provide an opportunity to inform therapeutic strategies that are more specific and thus have greater efficacy.

With this study, we suggest that neuron-type specific effects are vital to a full understanding of the effects of channelopathies at the level of neuronal firing. Furthermore, we highlight the use of modeling approaches to enable relatively fast and efficient insight into channelopathies.

## Data availability statement

The datasets presented in this study can be found in online repositories. The name of the repository and accession number can be found below: GitHub, https://github.com/nkoch1/LOFGOF2023.

## Author contributions

NAK, LS, UH, SL, and JB contributed to conception and design of the study. NAK performed simulation and wrote the first draft of the manuscript. NAK and LS analyzed simulation data. All authors wrote sections of the manuscript and contributed to manuscript revision, read, and approved the submitted version.
