# Supplementary material for: Loss or gain of function? Effects of ion channel mutations on neuronal firing depend on the neuron type
Source: Front Neurol. 2023 May 24;14:1194811. doi: 10.3389/fneur.2023.1194811 (PMC10244640; doi:10.3389/fneur.2023.1194811)
Supplement: Supplementary file 1 [file Data_Sheet_1.pdf]

Supplementary Material

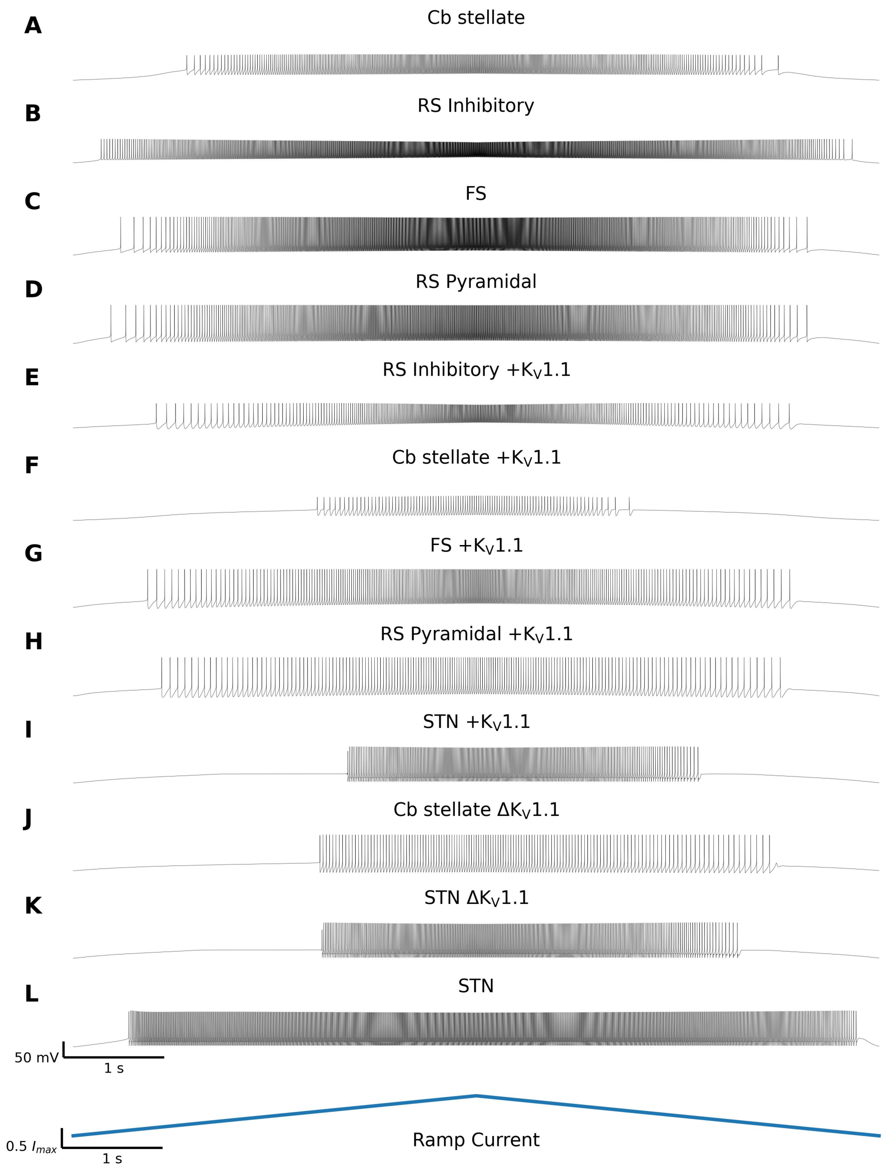

Figure S1: Diversity in Neuronal Model Firing Responses to a Current Ramp. Spike trains for Cb stellate **(A)**, RS inhibitory **(B)**, FS **(C)**, RS pyramidal **(D)**, RS inhibitory +K_V1.1 **(E)**, Cb stellate +K_V1.1 **(F)**, FS +K_V1.1 **(G)**, RS pyramidal +K_V1.1 **(H)**, STN +K_V1.1 **(I)**, Cb stellate ΔK_V1.1 **(J)**, STN ΔK_V1.1 **(K)**, and STN **(L)** neuron models in response to a slow ascending current ramp followed by the descending version of the current ramp (bottom). Models are ordered based on the qualitative fI curve sorting in Figure 1. The current at which firing begins in response to an ascending current ramp and the current at which firing ceases in response to a descending current ramp are depicted on the frequency current (fI) curves in Figure 1 for each model.
